# Age‐related changes in human skeletal muscle microstructure and architecture assessed by diffusion‐tensor magnetic resonance imaging and their association with muscle strength

**DOI:** 10.1111/acel.13851

**Published:** 2023-05-10

**Authors:** Donnie Cameron, David A. Reiter, Fatemeh Adelnia, Ceereena Ubaida‐Mohien, Christopher M. Bergeron, Seongjin Choi, Kenneth W. Fishbein, Richard G. Spencer, Luigi Ferrucci

**Affiliations:** ^1^ Norwich Medical School University of East Anglia Norwich UK; ^2^ C.J. Gorter MRI Center, Department of Radiology Leiden University Medical Center Leiden The Netherlands; ^3^ Translational Gerontology Branch, Intramural Research Program, National Institute on Aging National Institutes of Health Baltimore Maryland USA; ^4^ Emory University School of Medicine Atlanta Georgia USA; ^5^ Department of Radiology and Radiological Sciences, Vanderbilt University Institute of Imaging Science Vanderbilt University Medical Center Tennessee Nashville USA; ^6^ Laboratory of Clinical Investigation, Intramural Research Program, National Institute on Aging National Institutes of Health Baltimore Maryland USA; ^7^ Department of Neurology University of Maryland School of Medicine Baltimore Maryland USA

**Keywords:** aging, diffusion tensor imaging, muscle strength, sarcopenia, skeletal muscle fibers, thigh

## Abstract

Diffusion‐tensor magnetic resonance imaging (DT‐MRI) offers objective measures of muscle characteristics, providing insights into age‐related changes. We used DT‐MRI to probe skeletal muscle microstructure and architecture in a large healthy‐aging cohort, with the aim of characterizing age‐related differences and comparing these to muscle strength. We recruited 94 participants (43 female; median age = 56, range = 22–89 years) and measured microstructure parameters—fractional anisotropy (FA) and mean diffusivity (MD)—in 12 thigh muscles, and architecture parameters—pennation angle, fascicle length, fiber curvature, and physiological cross‐sectional area (PCSA)—in the rectus femoris (RF) and biceps femoris longus (BFL). Knee extension and flexion torques were also measured for comparison to architecture measures. FA and MD were associated with age (*β* = 0.33, *p* = 0.001, *R* ^2^ = 0.10; and *β* = −0.36, *p* < 0.001, *R* ^2^ = 0.12), and FA was negatively associated with Type I fiber proportions from the literature (*β* = −0.70, *p* = 0.024, and *R* ^2^ = 0.43). Pennation angle, fiber curvature, fascicle length, and PCSA were associated with age in the RF (*β* = −0.22, 0.26, −0.23, and −0.31, respectively; *p* < 0.05), while in the BFL only curvature and fascicle length were associated with age (*β* = 0.36, and −0.40, respectively; *p* < 0.001). In the RF, pennation angle and PCSA were associated with strength (*β* = 0.29, and 0.46, respectively; *p* < 0.01); in the BFL, only PCSA was associated with strength (*β* = 0.43; *p* < 0.001). Our results show skeletal muscle architectural changes with aging and intermuscular differences in the microstructure. DT‐MRI may prove useful for elucidating muscle changes in the early stages of sarcopenia and monitoring interventions aimed at preventing age‐associated microstructural changes in muscle that lead to functional impairment.

## Graphical abstract

We used diffusion‐tensor (DT)‐MRI to probe muscle microstructure and architecture in a healthy‐aging cohort. We observed age associations in muscle microstructure—fractional anisotropy and mean diffusivity—and architecture—pennation angle, fascicle length, fiber curvature, and physiological cross‐sectional area—and associations between architecture and strength. DT‐MRI may help elucidate muscle changes in sarcopenia and permit monitoring of interventions for mitigating functional impairment.

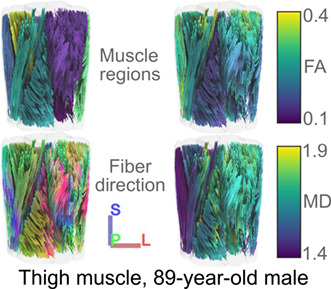

## 1 INTRODUCTION

With aging, skeletal muscles undergo progressive loss of volume and function, leading to impaired muscle strength, which is the hallmark of an age‐related disorder known as sarcopenia. Sarcopenia typically starts in middle age and then progresses at a highly variable rate between individuals (Cao & Morley, 2016). With the aging of the population, end‐stage sarcopenia is becoming a major societal problem, given its associations with falls, frailty, and death (Bischoff‐Ferrari et al., 2015; De Buyser et al., 2016). In older individuals, sarcopenia is currently diagnosed through assessment of lower extremity performance, including gait speed and the short physical performance battery (“Short Physical Performance Battery”, n.d.). When an individual demonstrates limited mobility, grip strength testing is then performed, followed by further diagnostic procedures, including imaging. However, in practice, cases are only identified when a patient reports or demonstrates signs of sarcopenia (Cruz‐Jentoft et al., 2019), at which point the symptoms are already so severe as to curtail function. A further obstacle to sarcopenia diagnosis is that global measures of physical function fail to capture local deficits and provide little information about the underlying mechanisms of muscle tissue derangement. These measures of performance can also be affected by motivation and compensatory motor strategies that may hide the progressive decline of strength (Guralnik & Ferrucci, 2003). In contrast, quantitative imaging offers objective measures of muscle architecture and quality and can be used to identify early derangements in skeletal muscle microstructure and architecture, monitor treatments' effects on these parameters, and ultimately link them to muscle function. Indeed, it is likely that quantitative changes in muscle may be detected at an early stage in individuals deemed “at risk” of sarcopenia, and treatments could be started when they are most likely to be effective.

Diffusion‐tensor magnetic resonance imaging (DT‐MRI)—a technique sensitive to the random motion, or diffusion, of water molecules within tissue—is proving increasingly useful for studying human skeletal muscle microstructure. Diffusion is measured in at least six directions, after which the results are combined mathematically to define the amount of diffusion along any direction. In skeletal muscle, three directions are of particular interest; recalling that muscle fibers have a roughly elliptical cross‐section, these are (i) along the fiber axis, and (ii) and (iii) along the major and minor axes of the cross‐section. Diffusivities along these three axes can be determined using DT‐MRI (Karampinos et al., 2009), and are reported as “diffusion eigenvalues” with the directions themselves referred to as “diffusion eigenvectors” due to the mathematical method through which they are determined. The average of these three values is referred to as the “mean diffusivity” (MD). The difference in diffusion values along these axes can be summarized by another DTI parameter, the “fractional anisotropy” (FA), which ranges from 0 for an isotropic medium, such as free water, to 1 for a maximally anisotropic medium, in which diffusion occurs along a single axis only. MD and FA are interrelated and have been shown to be sensitive to muscle histologic features, including fiber atrophy, denervation, inflammation, and cell de‐ and re‐generation, as summarized by Oudeman, Nederveen, et al. (2016).

In studies of muscle aging, fiber atrophy is of particular interest, with the decrease in muscle fiber size that occurs in sarcopenia contributing to the decline in muscle force generation (Lexell et al., 1983). However, only a few studies have investigated changes in diffusion metrics with age (Kermarrec et al., 2010; Lorbergs et al., 2015; Yanagisawa et al., 2009; Yoon et al., 2017), the most comprehensive being reported by Galbán et al. (2007), Sinha et al. (2015), and, more recently, Farrow et al. (2021). However, these studies were conducted in small groups with a relatively narrow age range, or, in the case of the study by Sinha et al. (2015), show data for only one sex.

Beyond conventional microstructural metrics, DT‐MRI can also be used to study the macroscopic arrangement of muscle fibers within a muscle, or muscle “architecture”: an important determinant of muscle force generation (Lieber & Fridén, 2000). DT‐MRI fiber tractography tracks the principal diffusion eigenvector, which reflects local muscle fiber orientation, across the muscle volume, permitting estimation of fiber pennation angle, curvature, fascicle length, and physiological cross‐sectional area (PCSA; Damon et al., 2002). These parameters are traditionally measured using ultrasound (Rutherford & Jones, 1992); however, DT‐MRI tractography has been validated against both ultrasound (Bolsterlee et al., 2015) and reference‐standard muscle dissection (Damon et al., 2002), and it has been shown to be repeatable (Heemskerk et al., 2010). Its advantages over ultrasound include the fact that measurements are made across an entire muscle or muscle group at once, and it avoids issues with tissue deformation resulting from the application of an ultrasound probe (Bolsterlee et al., 2015). While DT‐MRI muscle architecture measures are becoming both more widely used and more sophisticated, no in‐depth study of the effect of aging on these parameters yet exists. Muscle architecture is of particular interest in the context of aging, as fascicle length and pennation angle have been shown to decrease with age (Narici et al., 2003). Only Sinha et al. (2015) have applied muscle DT‐MRI tractography in the study of aging to date, comparing the lower‐leg muscles in small groups of younger and older women. A more comprehensive application in a larger cohort of male and female participants could elucidate age‐ and sex‐related effects in skeletal muscle microstructure and architecture, as measured by conventional DT‐MRI metrics and tractography, as well as the relationships of these parameters with muscle function.

In this work, we use DT‐MRI to probe thigh muscle microstructure and architecture in the large cohort of the GESTALT longitudinal study of aging. This represents the most in‐depth application of musculoskeletal DT‐MRI in aging to date, incorporating state‐of‐the‐art processing procedures and comprehensive muscle tractography analyses. To avoid the confounding effect of chronic diseases, whose prevalence typically increases with aging, participants enrolled in this study were characterized as healthy based on strict, objective criteria established via an in‐depth clinical protocol. In this select group, we aim to: (1) Characterize skeletal muscle microstructural differences with age and sex using DT‐MRI and systematically compare these to histology data from the literature; (2) Determine age‐ and sex‐related differences in skeletal muscle architecture and compare these characteristics to functional measures such as muscle strength.

## 2 RESULTS

Table 1 shows demographic and muscle strength data for the cohort studied here. All isokinetic and isometric torque measures were significantly greater in men than in women (*p* ≪ 0.001) and were negatively associated with age (*p* ≪ 0.001). Concentric, isokinetic torque measures obtained with angular velocities of 30°/s were chosen as the focus of muscle strength analyses because these slow contractions are strongly dependent on muscle function, whereas faster contractions are predominantly related to brain and nerve function.

**TABLE 1:** Demographic and muscle strength data for the participants included in this study.

	All	Female	Male	*p*‐Value
	(*n* = 94)	(*n* = 43, 46%)	(*n* = 51, 54%)	
Age, years	56.0 [36.4]	56.1 [30.0]	56.0 [39.0]	0.65
Race				0.12^a^
African‐American race, *N* (%)	11 (12)	2 (2)	9 (10)	
Caucasian race, *N* (%)	79 (84)	38 (40)	41 (44)	
Height, cm	171.1 (9.4)	164.1 (5.8)	177.2 (7.5)	≪0.001*
Weight, kg	75.5 (12)	67.5 (8.8)	82.4 (10)	≪0.001*
Body mass index, kg/m^2^	25.7 (2.6)	25.0 (2.8)	26.3 (2.3)	0.02*
Body surface area, m^2^	1.89 (0.19)	1.75 (0.13)	2.01 (0.15)	≪0.001*
Isometric knee extension torque 50°, Nm	134.2 [70.3]	107.2 (32.1)	164 (50.6)	≪0.001*
Isometric knee extension torque 70°, Nm	149.6 [81.7]	126.1 (42)	186.4 (68.6)	≪0.001*
Peak isokinetic knee extension torque at 30°/s, Nm	132 [74.4]	112.0 [62.7]	154.9 [72.1]	≪0.001*
Peak isokinetic knee extension torque at 180°/s, Nm	93.7 (33.1)	74.4 (25.1)	108.1 (31.1)	≪0.001*
Peak isokinetic knee flexion torque at 30°/s, Nm	62.9 (26)	48.0 [30.5]	73.2 [38.2]	<0.001*
Peak isokinetic knee flexion torque at 180°/s, Nm	56.2 [31.3]	46.9 (16.9)	67.9 (22.2)	≪0.001*

*Note*: Data are expressed as mean (standard deviation) if normally distributed or median [interquartile range] if not. Differences between men and women were assessed using Student's *t*‐tests or Mann–Whitney *U* tests, respectively. Sex differences in categorical data were assessed via chi‐squared tests.

^a^ Chi‐squared test *p*‐value.

* Statistically significant.

### 2.1 Differences in diffusion‐tensor MRI microstructure parameters by age and sex

**FIGURE 1:**
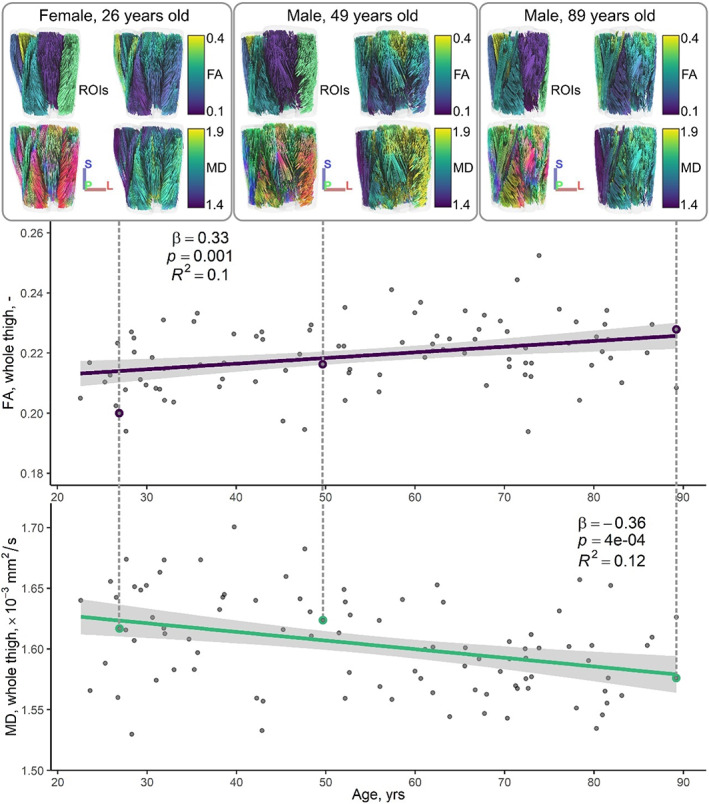
Representative tractography results (top row), and whole‐thigh fractional anisotropy (FA, middle row) and mean diffusivity (MD, bottom row) measures versus age. Dashed lines link the tractography examples to their corresponding FA and MD data points, which are highlighted by colored circles on the scatter plots. Tractography results are presented as anterior coronal views of the thigh showing, in clockwise order from the top left, muscle regions‐of‐interest (ROIs), tracts colored by their average FA and MD, respectively, and fiber tract directionality (red, left–right; green, anterior–posterior; blue, superior–inferior). In the scatter plots below, standardized linear regressions demonstrate associations between FA and age and MD and age, and regression lines, 95% confidence intervals, and regression statistics are shown.

Figure 1 illustrates the relationships between conventional DT‐MRI measures of muscle microstructure and age. FA and MD are expressed as weighted averages across all muscles of the thigh. Also shown are tractography examples from younger, middle‐aged, and older adults, where fibers are colored by region of interest (ROI), orientation, and FA and MD values.

Table S1 shows associations of FA and MD with age for each of the thigh muscles. The adductor longus (AL), adductor magnus (AM), gracilis (G), and vastus intermedius (VI) showed the strongest associations between FA and age, with *β* ranging from 0.28 to 0.49, *p* < 0.01. MD was most strongly associated with age in the AL, semimembranosus (SM), and vastus lateralis (VL), with *β* ranging from −0.31 to −0.48, *p* < 0.01.

Linear regression statistics for DT‐MRI microstructure measures versus age and sex are summarized in Table S2. Briefly, FA was positively associated with age and negatively associated with sex, being lower in men; MD was negatively associated with age and positively associated with sex, as was radial diffusivity, which represents diffusion perpendicular to muscle fibers. Axial diffusivity, which reflects diffusion along the long axis of muscle fibers, was negatively associated with age, but had no association with sex.

### 2.2 Intermuscular differences in diffusion‐tensor MRI microstructure parameters

**FIGURE 2:**
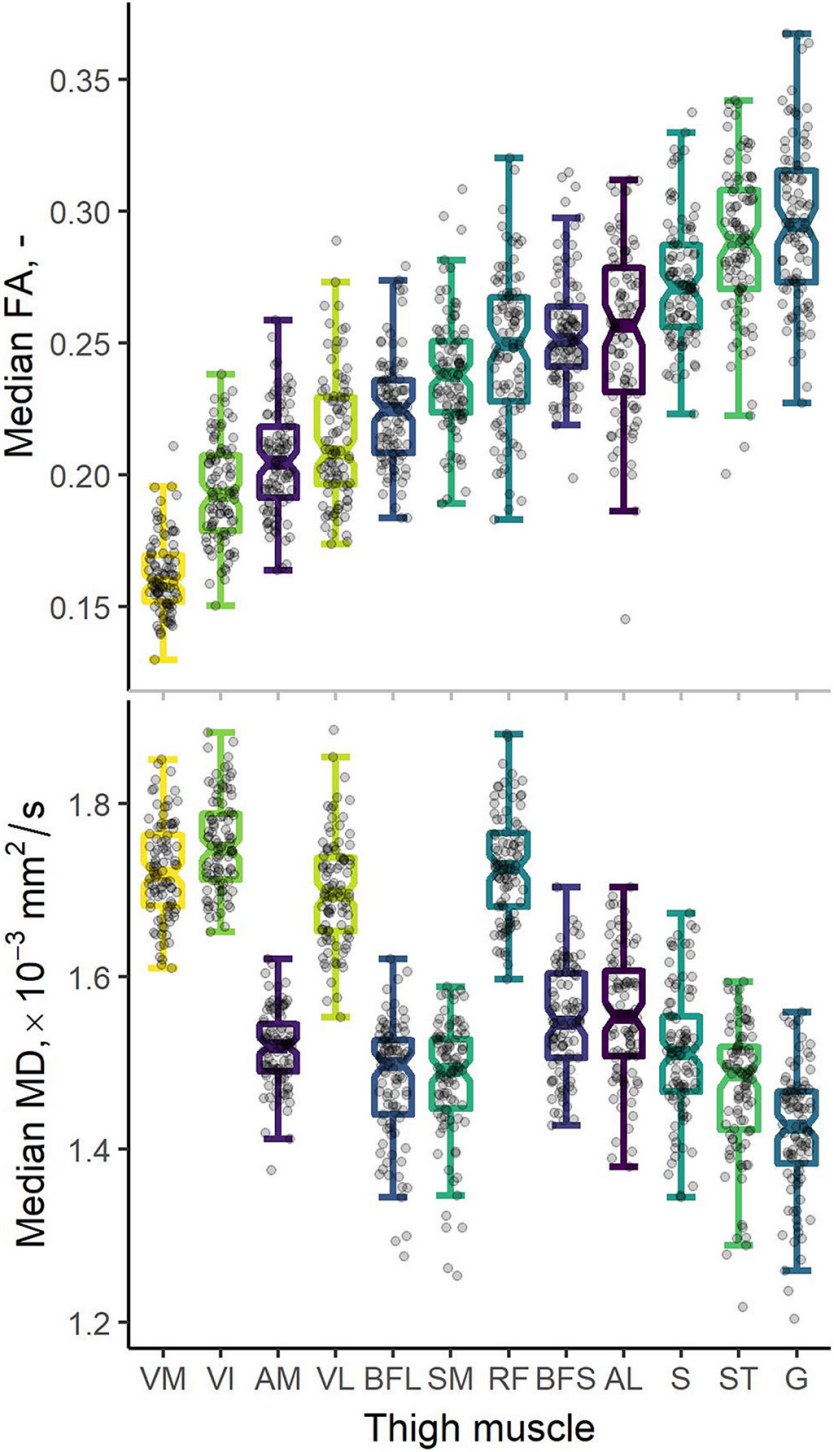
Boxplots showing fractional anisotropies (FA, top) and mean diffusivities (MD, bottom) derived from diffusion‐tensor MRI. Median region‐of‐interest (ROI) measures are shown for 12 muscles of the thigh: adductor longus (AL), adductor magnus (AM), biceps femoris long head (BFL), biceps femoris short head (BFS), gracilis (G), rectus femoris (RF), sartorius (S), semimembranosus (SM), semitendinosus (ST), vastus intermedius (VI), vastus lateralis (VL), and vastus medialis (VM). Boxplots represent median values by thick lines, with hinges indicating 25th and 75th percentiles and notches denoting confidence intervals around the median. The underlying raw data are represented by dots. Boxplots are sorted by increasing FA, highlighting a pattern of differences across the thigh where the quadriceps muscles tend to have lower FAs and higher MDs.

Welch's analysis of variance showed statistically significant differences in both median FA and median MD between muscles: *F*(11, 439) = 347.8 and *p* ≪ 0.001; and *F*(11, 439) = 334.7 and *p* ≪ 0.001, respectively. Post hoc Games‐Howell tests showed significant inter‐muscle differences in FA and MD, which are detailed in Tables S3 and S4, respectively. Most muscles showed characteristic FA and MD values that were significantly different from one other: out of 66 comparisons per parameter, only 7 indicated no difference in FA, and 11 showed no difference in MD. These differences are represented visually in Figure 2 and summary statistics are given in Table S5. The quadriceps muscles—the rectus femoris (RF), vastus medialis (VM), VL and VI—tended to have lower FAs and higher MDs as well as higher axial and radial diffusivities.

### 2.3 Diffusion‐tensor MRI microstructure parameters versus muscle fiber type

A literature search for articles reporting fiber‐type proportions in human leg muscles yielded three eligible autopsy studies (Edgerton et al., 1975; Nygaard & Sanchez, 1982; Vikne et al., 2012), and a fourth study where clear measures of fiber type were absent. Searching the reference lists of the remaining articles yielded two additional studies relevant to our comparisons (Garrett et al., 1984; Johnson et al., 1973). Together, these five articles provided Type I fiber ratios in infrequently‐biopsied muscles such as the RF, VI, VM, sartorius (S), AM, SM, semitendinosus (ST), and the biceps femoris long and short heads (BFL and BFS, respectively), as well as the commonly‐assessed VL muscle. Table S6 shows summary demographics and fiber‐type ratios for these studies.

Figure S1 shows plots of our median FA measures from muscles of the thigh versus the average of Type I muscle fiber proportions reported by Johnson et al. (1973) and Garrett et al. (1984) Median FA showed a statistically‐significant negative association with Type I fiber proportion (*β* = −0.70, *p* = 0.024, and *R* ^2^ = 0.43).

### 2.4 Differences in diffusion‐tensor MRI muscle architecture parameters by age and sex

All DT‐MRI datasets showed sufficient signal‐to‐noise ratios (SNRs) for accurate fascicle length, pennation angle, and fiber curvature calculation, exceeding the minimum threshold of 25 recommended by Froeling et al. (2013) (median = 63.2, range = 37.0–106.1).

**FIGURE 3:**
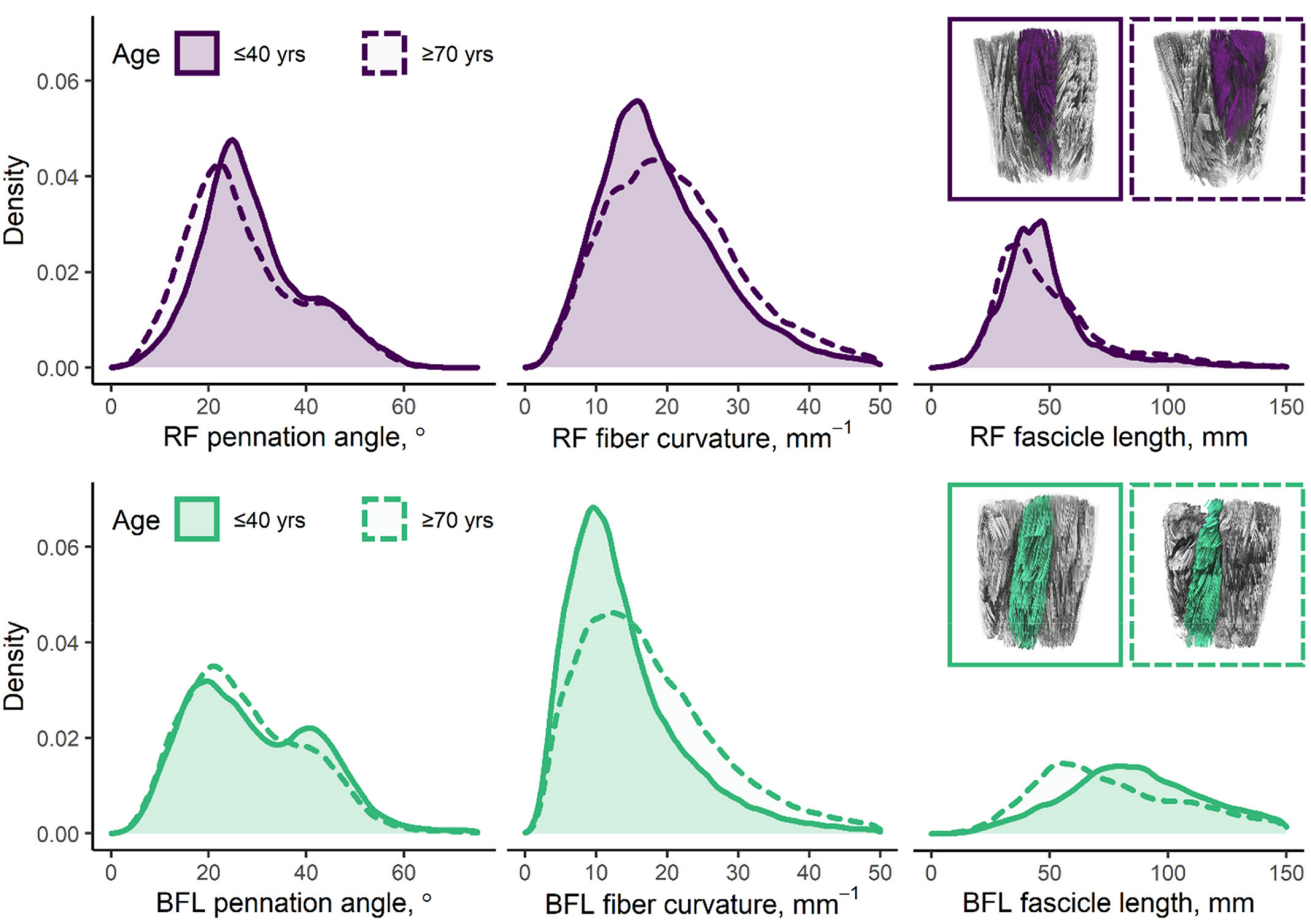
Density plots showing representative muscle architecture parameters in the rectus femoris (RF, top row) and biceps femoris longus (BFL, bottom row) muscles of the upper leg in two volunteers (both male, age = 30 and 78 years, BMI = 25.1 kg/m^2^ in both cases). Coronal tractography images, inset, highlight the structure of each muscle for both participants. In the RF, pennation angle and fascicle length tend to be lower in the older participant, while curvature is similar between participants. In the BFL, fascicle lengths are again lower in the older participant, but both pennation angle and curvature are higher. In both muscles, pennation angle appears to show a bimodal distribution, perhaps indicating different muscle compartments.

**FIGURE 4:**
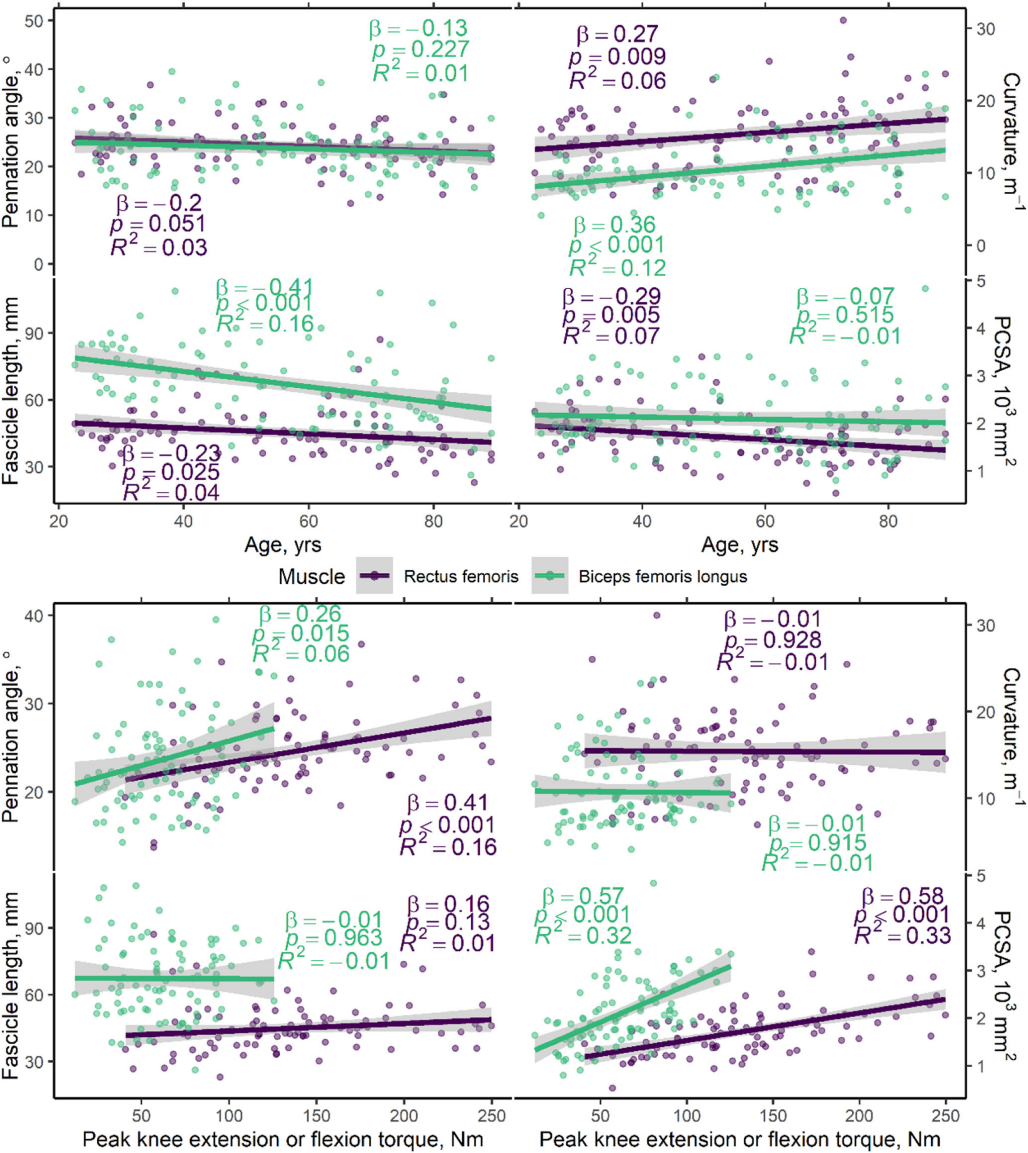
Scatter plots showing muscle architecture parameters versus age (top panel) and peak concentric, isokinetic knee extension, and flexion torques, obtained with an angular velocity of 30°/s (bottom panel). Results are shown separately for the rectus femoris (RF) and biceps femoris longus (BFL) muscles, with knee extension torques being reported for the former and flexion torques for the latter. Simple, unadjusted linear regressions show significant associations between curvature, fascicle length, and physiological cross‐sectional area (PCSA) and age, particularly in the RF. Positive associations are seen between pennation angle and torque and PCSA and torque, in both muscles. No clear trends are apparent between fiber curvature or fascicle length and torque. Regression lines and 95% confidence intervals are shown, along with unadjusted model statistics. See Table S3 for multiple regression statistics including sex as a covariate.

Figure 3 shows distributions of DT‐MRI muscle architecture measures in groups of younger and older participants. These distributions demonstrate two recognizable pennation‐angle peaks, or muscle architectural compartments, in both the RF and the BFL. Figure 4 (top panel) shows scatter plots and unadjusted regression statistics for these parameters versus age, in the whole cohort, again showing results for the RF and BFL.

Table 2 shows multiple regression statistics for DT‐MRI architecture parameters versus age and sex. The sampled volumes of the RF and BFL muscles in the mid‐to‐distal thigh were significantly associated with both age and sex. Pennation angle was significantly associated with age and sex in the RF, but not the BFL, where only sex was associated. Fiber curvature showed a significant association with age and sex in the RF, but only with age in the BFL (*β* = 0.36, *p* < 0.001; *p* = 0.060 for sex; adjusted *R* ^2^ = 0.13). Fascicle length was associated with age but not sex in the RF and BFL. Lastly, PCSA was significantly associated with age and sex in the RF, but only with sex in the BFL.

**TABLE 2:** Multiple linear regression models testing the association between diffusion‐tensor magnetic resonance imaging (DT‐MRI) architecture parameters and age (top section) and peak concentric knee extension or flexion torque at 30°/s (bottom section) in the rectus femoris and biceps femoris longus muscles, accounting for sex differences. All regression coefficients, *β*, are standardized.

	DT‐MRI architecture parameters vs. age and sex									
	Sampled volume		Pennation angle		Fiber curvature		Fascicle length		PCSA	
Rectus femoris	Adj. *R* ^2^ = 0.37		Adj. *R* ^2^ = 0.15		Adj. *R* ^2^ = 0.13		Adj. *R* ^2^ = 0.04		Adj. *R* ^2^ = 0.32	
	*β* [95% CI]	*p*‐Value	*β* [95% CI]	*p*‐Value	*β* [95% CI]	*p*‐Value	*β* [95% CI]	*p*‐Value	*β* [95% CI]	*p*‐Value
Age, years	−0.45 [−0.61, −0.29]	≪0.001*	−0.22 [−0.41, −0.03]	0.026*	0.26 [0.07, 0.45]	0.009*	−0.23 [−0.43, −0.03]	0.028*	−0.31 [−0.48, −0.14]	<0.001*
Sex	0.44 [0.28, 0.61]	≪0.001*	0.36 [0.17, 0.55]	<0.001*	0.28 [0.09, 0.47]	0.004*	−0.07 [−0.27, 0.13]	0.478	0.50 [0.33, 0.67]	≪0.001*

Biceps femoris	Adj. *R* ^2^ = 0.43		Adj. *R* ^2^ = 0.04		Adj. *R* ^2^ = 0.13		Adj. *R* ^2^ = 0.16		Adj. *R* ^2^ = 0.27	
	*β* [95% CI]	*p*‐Value	*β* [95% CI]	*p*‐Value	*β* [95% CI]	*p*‐Value	*β* [95% CI]	*p*‐Value	*β* [95% CI]	*p*‐Value
Age, years	−0.37 [−0.55, −0.19]	<0.001*	−0.13 [−0.34, 0.07]	0.191	0.36 [0.16, 0.55]	<0.001*	−0.40 [−0.59, −0.21]	<0.001*	−0.09 [−0.27, 0.09]	0.320
Sex	−0.38 [0.20, 0.56]	<0.001*	0.21 [0.01, 0.41]	0.043*	0.18 [−0.01, 0.37]	0.060	−0.11 [−0.30, 0.08]	0.244	0.53 [0.35, 0.70]	≪0.001*

	DT‐MRI architecture parameters vs knee extension or flexion strength and sex									
	Sampled volume		Pennation angle		Fiber curvature		Fascicle length		PCSA	
Rectus femoris	Adj. *R* ^2^ = 0.42		Adj. *R* ^2^ = 0.18		Adj. *R* ^2^ = 0.09		Adj. *R* ^2^ = 0.02		Adj. *R* ^2^ = 0.36	
	*β* [95% CI]	*p*‐Value	*β* [95% CI]	*p*‐Value	*β* [95% CI]	*p*‐Value	*β* [95% CI]	*p*‐Value	*β* [95% CI]	*p*‐Value
Knee extension torque, Nm	0.61 [0.42, 0.80]	≪0.001*	0.29 [0.08, 0.50]	0.008*	−0.21 [−0.44, 0.03]	0.084	0.23 [−0.01, 0.47]	0.055	0.46 [0.27, 0.66]	≪0.001*
Sex	0.10 [−0.09, 0.29]	0.295	0.19 [−0.02, 0.40]	0.080	0.39 [0.15, 0.63]	0.002*	−0.15 [−0.39, 0.09]	0.223	0.21 [0.02, 0.41]	0.032*

Biceps femoris	Adj. *R* ^2^ = 0.16		Adj. *R* ^2^ = 0.07		Adj. *R* ^2^ = 0.01		Adj. *R* ^2^ = −0.01		Adj. *R* ^2^ = 0.41	
	*β* [95% CI]	*p*‐Value	*β* [95% CI]	*p*‐Value	*β* [95% CI]	*p*‐Value	*β* [95% CI]	*p*‐Value	*β* [95% CI]	*p*‐Value
Knee flexion torque, Nm	0.18 [−0.03, 0.40]	0.098	0.19 [−0.03, 0.40]	0.094	−0.09 [−0.44, 0.03]	0.444	0.05 [−0.19, 0.28]	0.693	0.43 [0.25, 0.61]	≪0.001*
Sex	0.32 [0.10, 0.54]	0.004*	0.17 [−0.05, 0.39]	0.138	0.20 [−0.04, 0.43]	0.099	−0.13 [−0.37, 0.11]	0.277	0.35 [0.17, 0.53]	<0.001*

Abbreviation: PCSA, physiological cross‐sectional area.

* Statistically significant.

### 2.5 Muscle architecture measures from diffusion‐tensor MRI versus muscle strength

Scatter plots and unadjusted regression statistics for muscle architecture measures versus concentric knee extension and flexion torque in the RF and BFL are shown in Figure 4 (bottom panel).

Multiple regression statistics for DT‐MRI architecture parameters versus knee extension or flexion torque and sex are shown in Table 2. The sampled volume of the RF muscle was significantly associated with knee extension torque, but not sex, while the volume of the BFL was associated with sex, but not knee flexion torque. Pennation angle was associated with peak knee extension torque, but not sex, in the RF, while peak knee flexion torque in the BFL was not associated with pennation angle or sex. Muscle fiber curvature showed a significant association with sex but not peak knee extension torque in the RF muscle, whereas fiber curvature was neither associated with peak knee flexion torque nor sex in the BFL. There were no significant associations between fascicle length and peak torque or sex for either muscle. PCSA showed statistically‐significant associations with peak torque and sex in both muscles.

## 3 DISCUSSION

To our knowledge, ours is the first study to obtain DT‐MRI measures of skeletal muscle microstructure and architecture in a large aging cohort and relate muscle architecture measures to both aging and muscle strength. In our large cohort of participants, we found that FA tends to increase while MD decreases with aging, in the whole muscle volume of the thigh. In terms of muscle architecture measured in the RF and BFL muscles through tractography, we show that fiber pennation angle, fascicle length, and physiological cross‐sectional area tend to decrease with increasing age, while fiber curvature increases. Men had larger muscle volumes and PCSAs as well as higher pennation angles and fiber curvature than women but showed no significant differences in fascicle length. Lastly, we found that pennation angle and PCSA in the RF and the biceps femoris longus are positively associated with muscle strength, suggesting that these metrics capture characteristics of muscle tissue that are essential to strength generation. Importantly, this study was performed in a cohort of individuals who were free of chronic disease and were physically active but did not exercise more than 30 min/day. Therefore, it is unlikely that our findings are attributable to differences in health or physical activity.

### 3.1 DT‐MRI measures of skeletal muscle microstructure

Several studies have reported lower‐limb muscle microstructure measures from DT‐MRI in the context of aging, with varying results. Some report no change in FA and an increase in MD with age (Farrow et al., 2021; Sinha et al., 2015), while others report a decrease in FA and diffusion eigenvalues with age (Galbán et al., 2007), or no associations between FA and MD and age (Kermarrec et al., 2010). Results are contradictory, perhaps due to the small sample sizes studied. Our study is larger and better‐powered to detect microstructural changes with aging, though the associations with FA and MD we report here still show relatively small coefficients of determination. The source of much of the variability in skeletal muscle DT‐MRI metrics has not been explicitly determined to date; however, aspects such as intramuscular fat, perfusion, and fiber size are all known to influence FA and MD (Oudeman, Nederveen, et al., 2016). Indeed, the positive association between FA and age that we observe may be related to muscle fiber atrophy, particularly of the larger Type II fibers (Sinha et al., 2015), which leads to reduced fiber diameter. We also noted sex differences in DT‐MRI microstructural parameters, finding higher FA and lower MD values in women as compared to men. However, these results also conflict with others in the literature (Kermarrec et al., 2010; Oudeman, Nederveen, et al., 2016), and the origin of sex differences in DT‐MRI metrics remains unclear.

Our muscle‐specific analyses of microstructural associations with age highlighted specific muscles with stronger age associations, particularly the adductor muscles, which function as a postural stabilizer rather than a primary mover. While great emphasis has been placed on muscle strength and fiber type changes in primary movers with aging, there is evidence suggesting atrophy in stabilizing muscles could contribute more to fall risk in older adults (Daun & Kibele, 2019). Aging and disuse are associated with a fiber‐type conversion from faster Type II to slower Type I fibers that could be associated with denervation and reinnervation (Kelly et al., 2018) and cause the accumulation of very small fibers (Sonjak et al., 2019). These processes are expected to manifest on DT‐MRI as a change in FA, consistent with the results shown here. This may indicate a role for DT‐MRI microstructure metrics in the stabilizer muscles as therapeutic targets for reducing the risk of falls in older adults.

### 3.2 DT‐MRI microstructure metrics and muscle fiber type

We observed multiple FA and MD differences between muscles of the thigh, a phenomenon that has also been observed in the lower‐leg muscles (Sinha et al., 2006). Some have theorized that between‐muscle differences in FA relate to different fiber‐type proportions between muscles, as FA has been shown to be positively associated with the ratio of Type I to Type II muscle fibers (Scheel et al., 2013). To explore these differences, we collated literature measures of fiber‐type proportion in muscles of the thigh (Garrett et al., 1984; Johnson et al., 1973). In contrast to previous findings (Scheel et al., 2013), these measures were significantly, *negatively* associated with muscle‐specific FA measures from our study. Given that Type I fibers tend to have smaller diameters than Type II fibers, a higher proportion of Type I fibers would be expected to lead to more‐anisotropic diffusion and higher FA values. We, however, observed the opposite association, which perhaps suggests that our method is influenced by the known fiber‐type‐related differences in intracellular structures, such as transverse tubules, mitochondria, and glycogen stores, or that the extracellular matrix—the collagenous mesh within which muscle fibers are embedded—differs between fiber clusters of different types. Our spin‐echo DT‐MRI method probes a length scale of around 8 μm, and so cannot fully explore skeletal muscle's hierarchical structure, which spans length scales of 1–100 μm: from the tiny myofibrils, composed of actin and myosin myofilaments, to the larger Type II muscle fibers. Comparisons with stimulated‐echo DT‐MRI, which can probe longer length scales, may elucidate the origins of our findings.

### 3.3 DT‐MRI measures of skeletal muscle architecture

One of the strengths of this work is our detailed analysis of skeletal muscle architecture in the context of aging, sex, and muscle strength. Our observation that muscle fiber pennation angle, fascicle length, and PCSA tend to decrease with increasing age, particularly in the RF muscle, broadly agrees with localized ultrasound measurements of gastrocnemius medialis architecture (Narici et al., 2003). We also observed significantly greater muscle fiber curvatures in older participants, with greater curvature in the RF muscles of men than in women. Previous research has shown that fiber curvature may be detrimental to muscle function, creating pressure gradients between the convex and concave surfaces of muscle fibers, which may occlude blood flow (Miura et al., 2004). In our work, however, there was no significant association between curvature and muscle function, though the non‐significant trend to lower knee‐extension strength with higher RF fiber curvature (*β* = −0.21, *p* = 0.084) may merit further investigation in sarcopenic and frail individuals. In addition, blood flow in the same muscle could be measured in non‐invasive MRI perfusion studies to investigate the relationship between curvature and flow.

In individual muscles, our pennation angle measurements showed bi‐modal distributions, which may represent different muscle compartments. Previous work by Lansdown et al., (2007) showed similar findings in the tibialis anterior muscle of the lower leg, demonstrating larger pennation angles in the superior portion of the muscle than in the inferior portion. Using DT‐MRI over a whole muscle to measure the relative proportions of such compartments, and to characterize local pennation angle, may offer new insights into muscle structure and how it changes with age.

We showed that men had higher pennation angles, fiber curvature, and PCSAs than women, but no difference in fascicle length. These results are comparable with those of an ultrasound study by Behan et al. (2019), who showed that fascicle lengths in the BFL muscle did not differ between men and women. That study did not, however, show sex differences in pennation angle, though this may be due to the highly regional nature of ultrasound measurements.

Our muscle architecture measures from DT‐MRI were more strongly associated with age and function in the RF muscle than in the BFL muscle. This may be due to greater structural heterogeneity in the BFL, or it may relate to the different architectural constructions of the two muscles, with the RF being a pennate muscle, in contrast to the fusiform BFL. In pennate muscles, fibers are arranged obliquely to the muscle's line of action, placing more sarcomeres in parallel and leading to greater force production. In fusiform muscles, on the contrary, fibers tend to lie parallel to the muscle's line of action, placing more sarcomeres in series and leading, instead, to greater length excursions, permitting higher‐velocity contractions. The architecture of the pennate RF muscle, with its greater dynamic range of force production, may therefore result in a closer correlation between changes in muscle architecture and changes in muscle strength with aging. Narici et al. posit that sarcopenia involves a loss of sarcomeres both in parallel and in series, and they cite PCSA as a primary correlate in the loss of contractile force‐generating potential in older age: an observation that is supported by our results. Our important finding that DT‐MRI‐measured PCSA is positively associated with muscle strength in the RF and BFL muscles is also consistent with past ultrasound studies. Strasser et al. (2013) showed that quadriceps muscle pennation angles tend to be positively associated with maximum voluntary contraction in a study of groups of younger and older participants, with Pearson correlations ranging from 0.25 to 0.68. However, the correlation between pennation angle and maximum voluntary contraction was only statistically significant in the vastus intermedius of younger subjects. Training studies have also reported complementary findings. For example, Alonso‐Fernandez et al. (2019) showed that 8 weeks of eccentric training using reverse Nordic hamstring exercises led to increased pennation angle, cross‐sectional area, *and* fascicle length in the RF; these changes reverted after a detraining period. With our DT‐MRI approach, such training interventions could be monitored relatively easily, with the effects on different muscles and muscle regions being accessible within a single measurement. This also points to applications in sports science, where DT‐MRI architecture metrics such as pennation angle could serve as training targets. Further potential applications include to the deconditioning that occurs with immobilization and hospitalization, including related rehabilitation protocols, as well as to the range of inherited disorders of muscle metabolism and function.

### 3.4 Limitations

One limitation of our study is that we sampled only an 18‐cm portion of the thigh in our participants, rather than its whole superior–inferior extent. Considering the known proximal‐distal variation in muscle architecture (Kellis et al., 2010; Sinha et al., 2015), this may reduce our sensitivity to detect age‐ and sex‐related differences. The scan coverage also created challenges for matching literature data to in vivo DT‐MRI measures, as fiber‐type proportion varies both within and between muscles. To account for this, we used the closest‐matching muscle locations from the included studies. Another consideration is that our study cohort is composed entirely of healthy individuals, with no sarcopenic or frail participants, which may reduce the dynamic range of our results and therefore mask some associations. The links between frailty and DT‐MRI microstructure metrics have been explored by Farrow et al. (2021); however, no study to date has examined DT‐MRI architectural measures in explicitly sarcopenic or frail older adults. These metrics could be used as biomarkers to monitor the progression of sarcopenia and frailty and to evaluate exercise and pharmaceutical interventions for delaying their onset.

In conclusion, we have shown the utility of diffusion‐tensor MRI for exploring muscle architectural changes with aging across large muscle volumes, as well as for the exploration of intermuscular differences in skeletal muscle microstructure. Our approach may be further applied to studies of the progression of age‐related muscle architectural and microstructural changes into sarcopenia and explicit frailty, as well as applied to related therapeutic interventions.

## 4 EXPERIMENTAL PROCEDURES

### 4.1 Study population

We recruited 94 participants (43 female; median age = 56, range = 22–89 years) between April 2015 and January 2020 as part of the Genetic and Epigenetic Signatures of Translational Aging Laboratory Testing (GESTALT) study, conducted by the Clinical Research Branch of the National Institute on Aging at Harbor Hospital, Baltimore, MD. Through a clinical evaluation performed by expert research nurses that included medical history, a physical examination, and a comprehensive list of medical tests, participants were considered eligible if they: were aged 20 years or older; had no significant medical history or objective evidence of genetic, autoimmune, cardiovascular, renal, hepatic, musculoskeletal, or neurological disease; could perform normal activities of daily living and walk independently for at least 400 m without symptoms or shortness of breath; and were not undergoing chronic drug therapy, except antihypertensive monotherapy that effectively controlled blood pressure. The GESTALT protocol was reviewed and approved by the Institutional Review Board of the National Institute of Environmental Health Sciences. All participants received a comprehensive description of the study, including possible risks, and gave informed consent to participate.

### 4.2 Magnetic resonance imaging

MRI experiments were conducted on a Philips Achieva 3.0T X‐series system (Philips Healthcare) with a 32‐channel cardiac coil for signal reception. Subjects were positioned feet‐first with a 10‐cm bolster under their knees, to align the thigh muscles parallel to the bore, and the anterior and posterior elements of the array coil were fastened around both thighs using straps. Participants were then shifted laterally to place the left thigh close to isocenter. Hook‐and‐loop straps and padding were applied to minimize gross motion during the scan.

After localizers, a two‐point 2D chemical‐shift‐based water‐fat‐separation or “Dixon” sequence was applied in the left thigh to yield an anatomical reference. The distal edge of the axial slice stack was placed level with the insertion point of the vastus intermedius, giving good coverage of the central‐to‐distal portion of each thigh muscle. Scan parameters were: repetition time (TR) = 5.8 ms, echo time (TE) = 1.4 and 2.6 ms, flip angle = 6°, FOV = 256 mm × 228 mm, in‐plane resolution = 1 mm × 1 mm, 60 slices of 3‐mm thickness, and sensitivity encoding (SENSE) factor = 2.

DT‐MRI was applied over the same volume as the Dixon scan, using the following parameters: spin echo single‐shot echo‐planar imaging sequence, TR/TE = 3500/33 ms; field‐of‐view = 256 × 225 mm; 30 slices of 6‐mm thickness; in‐plane resolution = 2.56 mm × 2.61 mm; 8 averages; partial Fourier factor = 0.6; SENSE factor = 2; spectral adiabatic inversion recovery (SPAIR, inversion time = 70 ms, offset = 250 Hz) and slice‐select gradient reversal for fat suppression; 15 diffusion gradient directions, with *b* = 0 and 450 s/mm^2^ and a duration, *δ*, of 27 ms and interval, Δ, of 35 ms.

After DT‐MRI, a multi‐slice stack of B_0_ maps was collected to correct for susceptibility‐related distortions, and noise reference scans were collected for DT‐MRI SNR calculations.

The imaging protocol also included diffusion‐weighted imaging acquisitions, with *b*‐values ranging from 50 to 3000 s/mm^2^. These data are discussed elsewhere (Adelnia et al., 2019; Cameron et al., 2017).

### 4.3 Image processing and analysis

**FIGURE 5:**
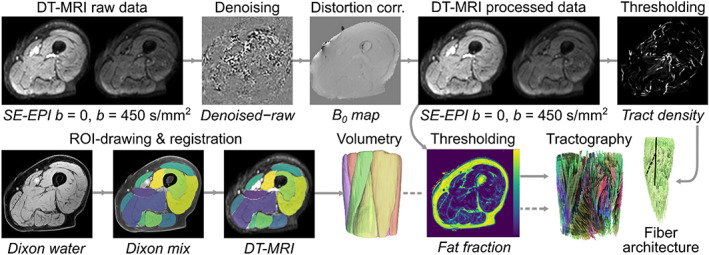
Schematic showing the diffusion tensor magnetic resonance imaging (DT‐MRI) data‐processing pipeline for this study. Representative axial spin‐echo echo planar imaging (SE‐EPI) DT‐MRI and Dixon data are shown for an 89‐year‐old male subject. The top row shows processing of the DT‐MRI raw data, including denoising and distortion correction, while the bottom row describes region‐of‐interest (ROI) drawing on Dixon images, registration of these ROIs to the DT‐MRI data, and the resulting volumetry. Tractography and fiber architecture determination is performed on the processed DT‐MRI data using muscle volumes from volumetry (dashed line) as seed regions and regions of high tract density (Oudeman, Mazzoli, et al., 2016) or high‐fat fraction as termination regions.

Our data processing strategy is briefly summarized in Figure 5. DT‐MRI data were post‐processed using a pipeline written in Python (v3.7, www.python.org) including denoising via an overcomplete local principal component analysis (PCA) filter (Manjón et al., 2013), and distortion‐ and eddy‐current correction via FSL's FUGUE and “eddy‐correct” functions, respectively (FMRIB, University of Oxford).

#### 4.3.1 SNR determination

SNR was estimated according to Yu et al. (2011), using a ROI drawn over the whole muscle area of the thickest part of the thigh.

#### 4.3.2 Region‐of‐interest delineation

Thigh muscle ROIs were drawn on Dixon images using 3D Slicer (v4, www.slicer.org) in the RF, VI, VM, VL, S, G, AM, AL, SM, ST, BFL, and BFS muscles. ROIs were traced at the center and proximal and distal ends of each muscle and were propagated across the whole muscle using a seed‐growing algorithm. To transform Dixon ROIs into the DT‐MRI image space, the images were aligned using sequential rigid, affine, and B‐spline transformations in elastix (v5.0.0, elastix.lumc.nl), which were then applied to each ROI.

#### 4.3.3 DT‐MRI microstructure metrics

Post‐processed data were exported to DSI Studio (2016‐01‐18 build, Fang‐Cheng Yeh, Carnegie Mellon University, PA, USA), where the diffusion‐tensor calculation was performed and MD and fractional anisotropy FA maps were generated. MD was defined as follows:

(1) $$\mathrm{MD}=\frac{\left(\lambda_{1}+\lambda_{2}+\lambda_{3}\right)}{3}=\bar{\lambda }$$

where *λ*_1_, *λ*_2_, and *λ*_3_ are the first, second, and third eigenvalues. FA was given by:

(2) $$\mathrm{FA}=\sqrt{\frac{3}{2}}\frac{\sqrt{{\left(\lambda_{1}-\bar{\lambda }\right)}^{2}+{\left(\lambda_{2}-\bar{\lambda }\right)}^{2}+{\left(\lambda_{3}-\bar{\lambda }\right)}^{2}}}{\sqrt{\lambda_{1}^{2}+\lambda_{2}^{2}+\lambda_{3}^{2}}}$$

Finally, axial and radial diffusion were defined as *λ*_1_ and (*λ*_2_ + *λ*_3_)/2, respectively.

#### 4.3.4 Fiber tracking

DT‐MRI eigenvalue and eigenvector data were used to perform fiber tractography for each muscle within DSI Studio. Thigh muscle ROIs served as seed regions for fiber tracking, while regions of high tract density (Oudeman, Mazzoli, et al., 2016), or proton density fat fraction >35%, served as terminative regions. The deterministic fiber‐tracking procedure used 2 mm seed‐spacing and 0.26 mm step length, and tracking was terminated if FA decreased below 0.1 or exceeded 0.5, or if the step‐to‐step angle change exceeded 10°. Tract‐specific DT‐MRI metrics were calculated by interpolation of the diffusion tensor at each point and tract coordinates and volumes were exported to MATLAB (2018a, MathWorks, Natick, MA, USA) to calculate architecture parameters.

#### 4.3.5 Fiber architecture metrics

Muscle fascicle lengths, and fiber pennation angles and curvatures were estimated in: (1) the RF, because age‐related atrophy of this muscle is representative of age‐related atrophy of the entire quadriceps (Trappe et al., 2001); and (2) the BFL, because this hamstring muscle is widely studied due its greater propensity for injury (Koulouris & Connell, 2003). Architectural parameters were determined as described by Bolsterlee et al. (2017) Muscle fiber tracts were transformed to a local coordinate system via PCA. Fascicle length was calculated as the length of a polynomial curve fitted to each tract, and pennation angle was computed as the angle between a fascicle's origin‐insertion line and the muscle's long axis determined from PCA. Fiber curvature was estimated using the Frenet‐Serret formula and muscle PCSAs were determined by dividing muscle volumes by their mean fascicle length.

### 4.4 Literature histology studies

To identify studies describing skeletal muscle fiber‐type proportions in the muscles of the thigh for comparison with our DT‐MRI data, we conducted a literature search in January 2022. Further details, including search terms, are given in the Supplemental Information.

### 4.5 Lower extremity muscle strength measurements

The strength of each participant's left leg was evaluated during isometric and concentric knee‐extension and flexion exercises using an isokinetic dynamometer (Biodex Multi‐Joint System‐PRO with Advantage Software v4X, Biodex Medical Systems, Inc.). Prior to measurements, the leg was weighed for gravity correction, and start‐stop angles were set to 90° and 30°. Maximum quadriceps muscle strength was defined as the highest of three consecutive measures of torque (N·m) determined from left‐leg knee extensor contractions at knee flexions of 50 and 70°. Torque itself was defined as the force generated during knee extension multiplied by the distance from the center of the knee to the point where the dynamometer was applied to the tibia. Participants also performed three sets of isokinetic concentric knee extensions and flexions at both 30°/s and 180°/s, where trials were separated by 15‐second rest intervals. Concentric, isokinetic peak torque was determined as the highest torque obtained throughout all trials. For all measurements, subjects were permitted several submaximal practice efforts prior to the recording of the best of three maximal efforts.

### 4.6 Statistics

All analyses were performed in R (v4.0, R Foundation for Statistical Computing). Parameters were checked for normality using Shapiro–Wilk tests, homogeneity of variance was determined using Brown‐Forsythe tests, and Welch analysis‐of‐variance was used to assess intermuscular differences in DT‐MRI metrics. Post‐hoc comparisons were performed with Tukey's range test if data showed equal variances or the Games‐Howell test if they did not, with multiple testing being considered. Sex differences were assessed using Student's *t*‐tests if data were normally distributed, or Mann–Whitney U tests if they were not. Associations between DT‐MRI parameters and age or muscle strength were evaluated via multiple linear regression. All regression analyses were adjusted for sex, which was coded as 0 for female and 1 for male, and we also tested for interactions between sex and the parameter of interest; however, we found no evidence for sex interactions with any of the tested parameters. Standardized variables were used for comparison of effect size, *β*, and *p* < 0.05 was considered statistically significant in all analyses.

## AUTHOR CONTRIBUTIONS

L.F. conceived the study; D.C., D.A.R., K.W.F., and R.G.S. designed the experiments; D.C., D.A.R., F.A, C.U., C.M.B., and S.C performed the experiments; D.C. analyzed the data; D.C. and L.F. drafted the manuscript. All authors interpreted the findings, commented on the manuscript, and approved the submitted version.

## CONFLICT OF INTEREST STATEMENT

The authors declare no competing financial interests.

## Supporting information


Appendix S1

## Data Availability

The data that support the findings of this study are available from the corresponding author upon reasonable request.
