# Supplementary material for: Age‐related changes in human skeletal muscle microstructure and architecture assessed by diffusion‐tensor magnetic resonance imaging and their association with muscle strength
Source: Aging Cell. 2023 May 10;22(7):e13851. doi: 10.1111/acel.13851 (PMC10352548; doi:10.1111/acel.13851)
Supplement: Supplementary file 1 — Appendix S1 [file ACEL-22-e13851-s001.docx]

**SUPPLEMENTARY EXPERIMENTAL PROCEDURES**

**Literature histology studies**

We searched the PubMed electronic database, excluding any articles not written in English. The search terms for PubMed were as follows: ("Muscle, Striated"[Mesh] OR "Muscle, Skeletal"[MeSH] OR (striated muscle[All Fields] OR striated muscles[All Fields] OR (skeletal muscle[All Fields] OR skeletal muscles[All Fields]))) AND (("fiber type"[All Fields] OR "fiber typing"[All Fields]) OR ("fibre type"[All Fields] OR "fibre typing"[All Fields])) AND "leg"[All Fields] AND ("cadaver" [All Fields] OR "autopsy"[All Fields]). *Ex vivo* studies were selected to exclude the large number of studies involving biopsies of the vastus lateralis muscle alone. The reference lists of each publication were also manually searched for other relevant articles.

When examining associations between *in vivo* fractional anisotropy (FA) measures from our study and fiber-type proportions from the literature, some assumptions were made to make the literature results more comparable. Namely, where fiber-type measures were reported as being from proximal or distal muscle locations, we used distal measures to better correspond to our mid-to-distal-thigh diffusion tensor MRI (DT-MRI) data. Further, where results were reported separately for surface and deep muscle locations, we used measures from deep muscle, as DT-MRI measures are less able to capture microstructural information about the surface of the muscle due to partial volume effects.

**SUPPLEMENTARY RESULTS**

|  | Fractional anisotropy × age | | | Mean diffusivity × age | | |
| --- | --- | --- | --- | --- | --- | --- |
|  | β [95% CI] | *R^2^* | *p*-value | β [95% CI] | *R^2^* | *p*-value |
| Adductor longus | 0.49 | 0.24 | ≪0.001* | -0.31 | 0.09 | 0.002* |
| Adductor magnus | 0.28 | 0.07 | 0.006* | 0.03 | -0.01 | 0.794 |
| Biceps femoris long head | 0.03 | -0.01 | 0.748 | 0.07 | -0.01 | 0.482 |
| Biceps femoris short head | -0.15 | 0.01 | 0.136 | -0.22 | 0.04 | 0.032* |
| Gracilis | 0.29 | 0.07 | 0.005* | -0.24 | 0.05 | 0.022* |
| Rectus femoris | -0.11 | 0 | 0.282 | -0.07 | -0.01 | 0.487 |
| Sartorius | 0.19 | 0.02 | 0.07 | -0.13 | 0.01 | 0.206 |
| Semimembranosus | 0.03 | -0.01 | 0.771 | -0.48 | 0.22 | ≪0.001* |
| Semitendinosus | 0.15 | 0.01 | 0.158 | -0.18 | 0.02 | 0.087 |
| Vastus intermedius | 0.35 | 0.12 | <0.001* | -0.18 | 0.02 | 0.088 |
| Vastus lateralis | 0.02 | -0.01 | 0.864 | -0.32 | 0.09 | 0.002* |
| Vastus medialis | 0.24 | 0.05 | 0.019* | -0.24 | 0.05 | 0.021* |

**Supplemental Table S1.** *Standardized regression parameters showing associations between diffusion parameters and age for muscles of the thigh. * = statistically significant*

|  | | DT-MRI microstructure parameters vs age and sex | | | | | | | |
| --- | --- | --- | --- | --- | --- | --- | --- | --- | --- |
|  | FA Adj. *R^2^* = 0.19 | | | MD  Adj. *R^2^* = 0.17 | | AD  Adj. *R^2^* = 0.06 | | RD  Adj. *R^2^* = 0.24 | |
|  | β [95% CI] | | *p*-value | β [95% CI] | *p­*-value | β [95% CI] | *p*-value | β [95% CI] | *p*-value |
| Age, yrs | 0.34 [0.16, 0.53] | | <0.001* | −0.37 [−0.56, −0.18] | <0.001* | −0.27 [−0.47, −0.07] | 0.009* | −0.41 [−0.59, −0.23] | <0.001* |
| Sex | −0.32 [−0.50, −0.13] | | 0.001* | 0.24 [0.05, 0.43] | 0.013* | 0.07 [−0.13, 0.27] | 0.470 | 0.31 [0.13, 0.49] | 0.001* |

**Supplemental Table S2.** *Multiple linear regression models testing the association between diffusion-tensor magnetic resonance imaging (DT-MRI) microstructure parameters and age, accounting for sex differences. All regression coefficients, β, are standardized. * = statistically significant. AD = axial diffusivity, FA = fractional anisotropy, MD = mean diffusivity, RD = radial diffusivity. DT-MRI = diffusion-tensor magnetic resonance imaging.*

|  | Muscle 1 | Muscle 2 | Estimate [95% CI] | Adj. *p*-value |
| --- | --- | --- | --- | --- |
| 1 | AL | AM | -0.0492 [-0.062, -0.0364] | ≪0.00* |
| 2 | AL | BFS | ≪0.001 0336 [-0.0131, 0.0131] | 1.000* |
| 3 | AL | BFL | -0.0301 [-0.043, -0.0171] | ≪0.001* |
| 4 | AL | G | 0.0411 [0.026, 0.0561] | ≪0.001* |
| 5 | AL | RF | -0.00654 [-0.0212, 0.00815] | 0.946 |
| 6 | AL | S | 0.0189 [0.00542, 0.0324] | ≪0.001* |
| 7 | AL | SM | -0.0158 [-0.029, -0.00251] | 0.006* |
| 8 | AL | ST | 0.0335 [0.0188, 0.0481] | ≪0.001* |
| 9 | AL | VI | -0.061 [-0.0737, -0.0483] | ≪0.001* |
| 10 | AL | VL | -0.0408 [-0.0545, -0.0271] | ≪0.001* |
| 11 | AL | VM | -0.0927 [-0.105, -0.0807] | ≪0.001* |
| 12 | AM | BFS | 0.0492 [0.0392, 0.0592] | ≪0.001* |
| 13 | AM | BFL | 0.0191 [0.00933, 0.0289] | ≪0.001* |
| 14 | AM | G | 0.0902 [0.0777, 0.103] | ≪0.001* |
| 15 | AM | RF | 0.0426 [0.0306, 0.0547] | ≪0.001* |
| 16 | AM | S | 0.0681 [0.0576, 0.0785] | ≪0.001* |
| 17 | AM | SM | 0.0334 [0.0232, 0.0436] | ≪0.001* |
| 18 | AM | ST | 0.0826 [0.0707, 0.0946] | ≪0.001* |
| 19 | AM | VI | -0.0118 [-0.0212, -0.00242] | 0.003* |
| 20 | AM | VL | 0.00836 [-0.00238, 0.0191] | 0.300 |
| 21 | AM | VM | -0.0435 [-0.0519, -0.0352] | ≪0.001* |
| 22 | BFS | BFL | -0.0301 [-0.0403, -0.0199] | ≪0.001* |
| 23 | BFS | G | 0.041 [0.0282, 0.0538] | ≪0.001* |
| 24 | BFS | RF | -0.00658 [-0.0189, 0.00574] | 0.832 |
| 25 | BFS | S | 0.0189 [0.00806, 0.0297] | ≪0.001* |
| 26 | BFS | SM | -0.0158 [-0.0263, -0.00527] | ≪0.001* |
| 27 | BFS | ST | 0.0334 [0.0211, 0.0457] | ≪0.001* |
| 28 | BFS | VI | -0.061 [-0.0708, -0.0513] | ≪0.001* |
| 29 | BFS | VL | -0.0409 [-0.0519, -0.0298] | ≪0.001* |
| 30 | BFS | VM | -0.0928 [-0.102, -0.0839] | ≪0.001* |
| 31 | BFL | G | 0.0711 [0.0584, 0.0838] | ≪0.001* |
| 32 | BFL | RF | 0.0235 [0.0113, 0.0357] | ≪0.001* |
| 33 | BFL | S | 0.0489 [0.0383, 0.0596] | ≪0.001* |
| 34 | BFL | SM | 0.0143 [0.00393, 0.0246] | 0.001* |
| 35 | BFL | ST | 0.0635 [0.0514, 0.0757] | ≪0.001* |
| 36 | BFL | VI | -0.031 [-0.0406, -0.0214] | ≪0.001* |
| 37 | BFL | VL | -0.0108 [-0.0217, ≪0.001 149] | 0.057 |
| 38 | BFL | VM | -0.0627 [-0.0713, -0.0541] | ≪0.001* |
| 39 | G | RF | -0.0476 [-0.062, -0.0332] | ≪0.001* |
| 40 | G | S | -0.0222 [-0.0353, -0.009] | ≪0.001* |
| 41 | G | SM | -0.0568 [-0.0698, -0.0439] | ≪0.001* |
| 42 | G | ST | -0.00759 [-0.022, 0.0068] | 0.844 |
| 43 | G | VI | -0.102 [-0.114, -0.0897] | ≪0.001* |
| 44 | G | VL | -0.0819 [-0.0953, -0.0685] | ≪0.001* |
| 45 | G | VM | -0.134 [-0.145, -0.122] | ≪0.001* |
| 46 | RF | S | 0.0254 [0.0127, 0.0381] | ≪0.001* |
| 47 | RF | SM | -0.00923 [-0.0217, 0.00325] | 0.378 |
| 48 | RF | ST | 0.04 [0.026, 0.054] | ≪0.001* |
| 49 | RF | VI | -0.0545 [-0.0663, -0.0426] | ≪0.001* |
| 50 | RF | VL | -0.0343 [-0.0472, -0.0214] | ≪0.001* |
| 51 | RF | VM | -0.0862 [-0.0973, -0.0751] | ≪0.001* |
| 52 | S | SM | -0.0347 [-0.0456, -0.0237] | ≪0.001* |
| 53 | S | ST | 0.0146 [0.0019, 0.0272] | 0.010* |
| 54 | S | VI | -0.0799 [-0.0902, -0.0696] | ≪0.001* |
| 55 | S | VL | -0.0597 [-0.0712, -0.0482] | ≪0.001* |
| 56 | S | VM | -0.112 [-0.121, -0.102] | ≪0.001* |
| 57 | SM | ST | 0.0492 [0.0368, 0.0617] | ≪0.001* |
| 58 | SM | VI | -0.0452 [-0.0552, -0.0352] | ≪0.001* |
| 59 | SM | VL | -0.0251 [-0.0363, -0.0138] | ≪0.001* |
| 60 | SM | VM | -0.0769 [-0.086, -0.0679] | ≪0.001* |
| 61 | ST | VI | -0.0945 [-0.106, -0.0826] | ≪0.001* |
| 62 | ST | VL | -0.0743 [-0.0872, -0.0614] | ≪0.001* |
| 63 | ST | VM | -0.126 [-0.137, -0.115] | ≪0.001* |
| 64 | VI | VL | 0.0202 [0.00962, 0.0308] | ≪0.001* |
| 65 | VI | VM | -0.0317 [-0.0399, -0.0236] | ≪0.001* |
| 66 | VL | VM | -0.0519 [-0.0616, -0.0422] | ≪0.001* |

**Supplemental Table S3.** *Games-Howell post-hoc tests showing between-muscle differences in median fractional anisotropy. * = statistically significant. AL = adductor longus,* *AM = adductor magnus, BFL = biceps femoris long head, BFS = biceps femoris short head, G = gracilis, RF = rectus femoris, S = sartorius, SM = semimembranosus, ST = semitendinosus, VI = vastus intermedius, VL = vastus lateralis, VM = vastus medialis.*

|  | Muscle 1 | Muscle 2 | Estimate [95% CI] | Adj. *p*-value |
| --- | --- | --- | --- | --- |
| 1 | AL | AM | -0.0373 [-0.067, -0.00761] | 0.003* |
| 2 | AL | BFS | -0.00566 [-0.0388, 0.0274] | 1.000 |
| 3 | AL | BFL | -0.074 [-0.109, -0.0392] | ≪0.001* |
| 4 | AL | G | -0.133 [-0.168, -0.0971] | ≪0.001* |
| 5 | AL | RF | 0.174 [0.141, 0.207] | ≪0.001* |
| 6 | AL | S | -0.0436 [-0.0796, -0.0075] | 0.005* |
| 7 | AL | SM | -0.0731 [-0.108, -0.0384] | ≪0.001* |
| 8 | AL | ST | -0.0847 [-0.121, -0.0481] | ≪0.001* |
| 9 | AL | VI | 0.199 [0.168, 0.23] | ≪0.001* |
| 10 | AL | VL | 0.145 [0.111, 0.178] | ≪0.001* |
| 11 | AL | VM | 0.169 [0.137, 0.201] | ≪0.001* |
| 12 | AM | BFS | 0.0317 [0.00555, 0.0578] | 0.005* |
| 13 | AM | BFL | -0.0367 [-0.065, -0.00841] | 0.002* |
| 14 | AM | G | -0.0954 [-0.125, -0.0661] | ≪0.001* |
| 15 | AM | RF | 0.211 [0.185, 0.237] | ≪0.001* |
| 16 | AM | S | -0.00625 [-0.0361, 0.0236] | 1.000 |
| 17 | AM | SM | -0.0358 [-0.064, -0.00763] | 0.002* |
| 18 | AM | ST | -0.0474 [-0.0779, -0.0168] | ≪0.001* |
| 19 | AM | VI | 0.236 [0.212, 0.26] | ≪0.001* |
| 20 | AM | VL | 0.182 [0.156, 0.208] | ≪0.001* |
| 21 | AM | VM | 0.206 [0.182, 0.231] | ≪0.001* |
| 22 | BFS | BFL | -0.0683 [-0.1, -0.0365] | ≪0.001* |
| 23 | BFS | G | -0.127 [-0.16, -0.0944] | ≪0.001* |
| 24 | BFS | RF | 0.179 [0.149, 0.209] | ≪0.001* |
| 25 | BFS | S | -0.0379 [-0.0711, -0.00467] | 0.011* |
| 26 | BFS | SM | -0.0675 [-0.0992, -0.0357] | ≪0.001* |
| 27 | BFS | ST | -0.079 [-0.113, -0.0452] | ≪0.001* |
| 28 | BFS | VI | 0.205 [0.177, 0.233] | ≪0.001* |
| 29 | BFS | VL | 0.15 [0.12, 0.181] | ≪0.001* |
| 30 | BFS | VM | 0.175 [0.146, 0.203] | ≪0.001* |
| 31 | BFL | G | -0.0588 [-0.0932, -0.0243] | ≪0.001* |
| 32 | BFL | RF | 0.248 [0.216, 0.28] | ≪0.001* |
| 33 | BFL | S | 0.0304 [-0.00448, 0.0654] | 0.155 |
| 34 | BFL | SM | ≪0.001 897 [-0.0326, 0.0344] | 1.000 |
| 35 | BFL | ST | -0.0107 [-0.0462, 0.0248] | 0.998 |
| 36 | BFL | VI | 0.273 [0.243, 0.303] | ≪0.001* |
| 37 | BFL | VL | 0.219 [0.187, 0.251] | ≪0.001* |
| 38 | BFL | VM | 0.243 [0.213, 0.274] | ≪0.001* |
| 39 | G | RF | 0.306 [0.274, 0.339] | ≪0.001* |
| 40 | G | S | 0.0892 [0.0535, 0.125] | ≪0.001* |
| 41 | G | SM | 0.0597 [0.0253, 0.094] | ≪0.001* |
| 42 | G | ST | 0.0481 [0.0118, 0.0844] | 0.001* |
| 43 | G | VI | 0.332 [0.301, 0.363] | ≪0.001* |
| 44 | G | VL | 0.277 [0.244, 0.31] | ≪0.001* |
| 45 | G | VM | 0.302 [0.27, 0.333] | ≪0.001* |
| 46 | RF | S | -0.217 [-0.251, -0.184] | ≪0.001* |
| 47 | RF | SM | -0.247 [-0.279, -0.215] | ≪0.001* |
| 48 | RF | ST | -0.258 [-0.292, -0.225] | ≪0.001* |
| 49 | RF | VI | 0.0253 [-0.00294, 0.0535] | 0.127 |
| 50 | RF | VL | -0.0291 [-0.0594, 0.00125] | 0.074 |
| 51 | RF | VM | -0.00466 [-0.0333, 0.024] | 1.000 |
| 52 | S | SM | -0.0295 [-0.0644, 0.00529] | 0.185 |
| 53 | S | ST | -0.0411 [-0.0779, -0.00439] | 0.014* |
| 54 | S | VI | 0.243 [0.211, 0.274] | ≪0.001* |
| 55 | S | VL | 0.188 [0.155, 0.222] | ≪0.001* |
| 56 | S | VM | 0.213 [0.181, 0.245] | ≪0.001* |
| 57 | SM | ST | -0.0116 [-0.047, 0.0238] | 0.995 |
| 58 | SM | VI | 0.272 [0.242, 0.302] | ≪0.001* |
| 59 | SM | VL | 0.218 [0.186, 0.25] | ≪0.001* |
| 60 | SM | VM | 0.242 [0.212, 0.273] | ≪0.001* |
| 61 | ST | VI | 0.284 [0.251, 0.316] | ≪0.001* |
| 62 | ST | VL | 0.229 [0.195, 0.263] | ≪0.001* |
| 63 | ST | VM | 0.254 [0.221, 0.286] | ≪0.001* |
| 64 | VI | VL | -0.0543 [-0.0828, -0.0259] | ≪0.001* |
| 65 | VI | VM | -0.0299 [-0.0566, -0.00327] | 0.014* |
| 66 | VL | VM | 0.0244 [-0.00449, 0.0533] | 0.190 |

**Supplemental Table S4.** *Games-Howell post-hoc tests showing between-muscle differences in median mean diffusivity. * = statistically significant. AL = adductor longus,* *AM = adductor magnus, BFL = biceps femoris long head, BFS = biceps femoris short head, G = gracilis, RF = rectus femoris, S = sartorius, SM = semimembranosus, ST = semitendinosus, VI = vastus intermedius, VL = vastus lateralis, VM = vastus medialis.*

|  | Fractional anisotropy | Mean diffusivity, ×10^-3^ mm^2^/s | Axial diffusivity, ×10^-3^ mm^2^/s | Radial diffusivity, ×10^-3^ mm^2^/s |
| --- | --- | --- | --- | --- |
| Adductor longus | 0.25 (0.03) | 1.55 (0.07) | 1.99 (0.09) | 1.34 (0.08) |
| Adductor magnus | 0.21 (0.02) | 1.52 (0.04) | 1.86 (0.06) | 1.34 (0.05) |
| Biceps femoris long head | 0.22 (0.02) | 1.48 (0.07) | 1.86 (0.08) | 1.29 (0.07) |
| Biceps femoris short head | 0.25 (0.02) | 1.55 (0.06) | 1.99 (0.07) | 1.33 (0.06) |
| Gracilis | 0.30 (0.03) | 1.42 (0.07) | 1.91 (0.07) | 1.17 (0.08) |
| Rectus femoris | 0.25 (0.03) | 1.73 (0.06) | 2.20 (0.11) | 1.49 (0.06) |
| Sartorius | 0.27 (0.02) | 1.51 (0.07) | 1.99 (0.10) | 1.27 (0.07) |
| Semimembranosus | 0.24 (0.02) | 1.48 (0.07) | 1.88 (0.08) | 1.28 (0.07) |
| Semitendinosus | 0.29 (0.03) | 1.47 (0.08) | 1.97 (0.08) | 1.22 (0.08) |
| Vastus intermedius | 0.19 (0.02) | 1.75 (0.05) | 2.11 (0.07) | 1.57 (0.05) |
| Vastus lateralis | 0.21 (0.02) | 1.70 (0.06) | 2.08 (0.10) | 1.50 (0.06) |
| Vastus medialis | 0.16 (0.01) | 1.72 (0.06) | 2.03 (0.07) | 1.57 (0.06) |

**Supplemental Table S5.** *Diffusion parameter summary statistics for muscles of the thigh. Data are expressed as mean (standard deviation).*

**Supplemental Table S6.** Proportion of Type I muscle fibers in muscles of the thigh as reported in cadaver studies in the literature.

| Article (year) | Pre-mortem age, yrs | Cohort, *N* male/female | Type I muscle fiber proportion (%) | | | | | | | | | |
| --- | --- | --- | --- | --- | --- | --- | --- | --- | --- | --- | --- | --- |
|  |  |  | AM | BFL | BFS | RF | S | SM | ST | VI | VL | VM |
| Johnson et al. (1973)^1^ | 18−30 | 6M | 63.3 | 66.9* | − | 42 | 49.6 | − | − | − | 46.9 | 61.5 |
| Edgerton et al. (1975)^2^ | 21−83 | 22M, 10F | − | − | − | − | − | − | − | 47 | 32 | − |
| Nygaard et al. (1982)^3^ | 72−88 | 5M | − | − | − | − | − | − | − | − | 45 | − |
| Garrett et al. (1984)†^4^ | 37−76 | 7M, 3F | 55.2 | 46.2 | 40.8 | 42.3 | − | 49.2 | 39.6 | 54.3 | 45.5 | 50.6 |
| Vikne et al. (2012)^5^ | 18−65 | 11M, 1F | − | − | − | − | − | − | − | − | 31.9 | − |

*Note. AM = adductor magnus, BFL = biceps femoris long head, BFS = biceps femoris short head, RF = rectus femoris, S = sartorius, SM = semimembranosus, ST = semitendinosus, VI = vastus intermedius, VL = vastus lateralis, VM = vastus medialis. *Head and location not specified, but likely refers to the larger, more superficial ‘long’ head. †Hamstring results are reported as being from proximal or distal muscle locations – for comparison to our work, we present distal measures here. Further, where results were reported separately for surface and deep muscle locations, we have used measures from deep muscle. The Type I fiber proportions used for plotting Supplemental Figure S1 were obtained by averaging measures from the Johnson and Garrett articles.*


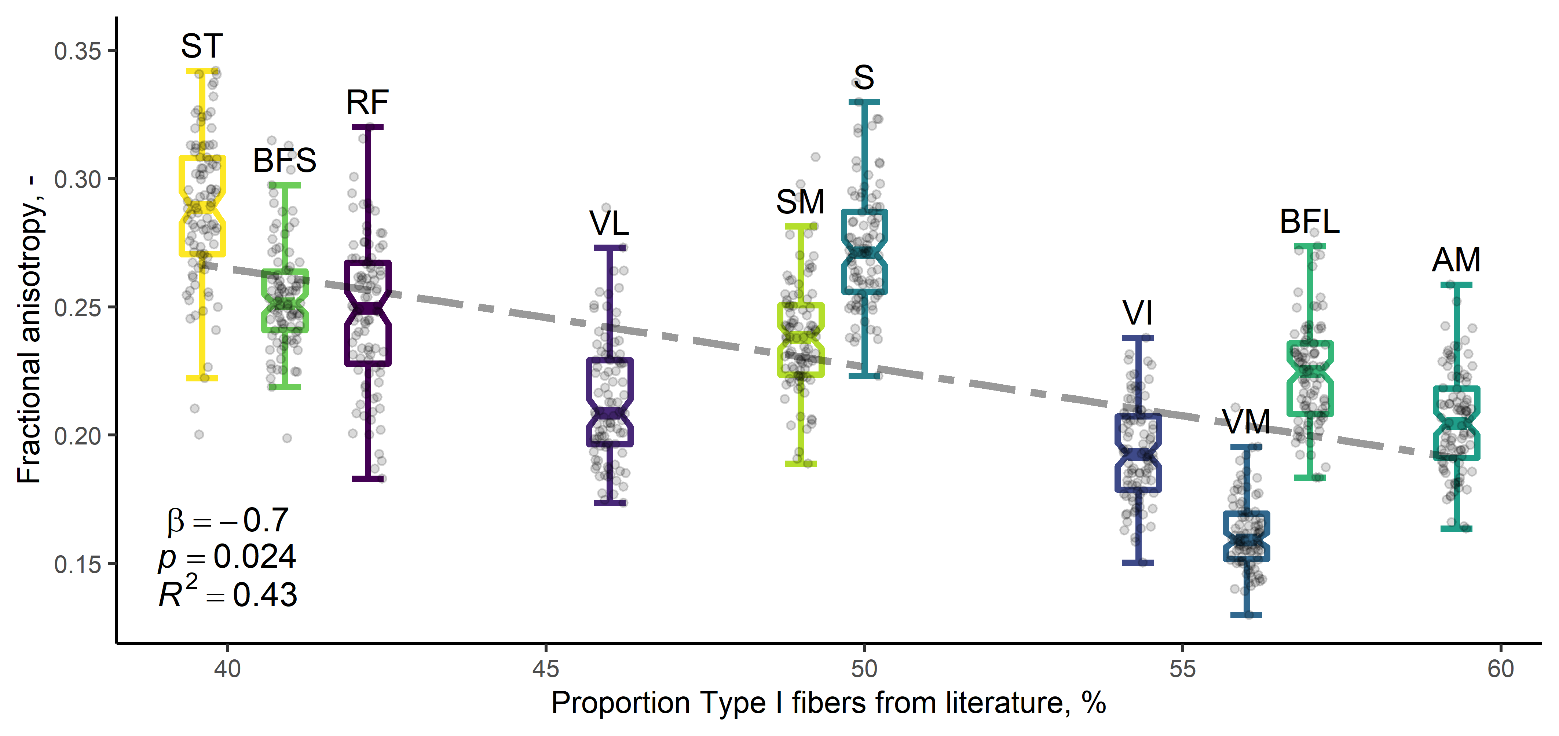


***Supplemental Figure S1.*** *Boxplots showing the relationship between fractional anisotropy (FA) from diffusion-tensor MRI and fiber-type proportion in skeletal muscle. Median per-muscle FA values from this study are plotted against the literature-reported Type I fiber proportions for these muscles, averaged together from autopsy studies by Johnson et al.^1^ and Garrett et al.^4^, where available. See Supplemental Tables S5 and S6. The dashed regression line highlights the statistically-significant trend whereby muscles with a higher proportion of Type I fibers tend to show lower FA values. Regression model statistics are also shown. AM = adductor magnus, BFL = biceps femoris long head, BFS = biceps femoris short head, RF = rectus femoris, S = sartorius, SM = semimembranosus, ST = semitendinosus, VI = vastus intermedius, VL = vastus lateralis, VM = vastus medialis.*

**REFERENCES**

1. Johnson MA, Polgar J, Weightman D, Appleton D. Data on the distribution of fibre types in thirty-six human muscles: an autopsy study. *Journal of the neurological sciences.* 1973;18(1):111-129. doi:10.1016/0022-510x(73)90023-3

2. Edgerton VR, Smith JL, Simpson DR. Muscle fibre type populations of human leg muscles. *The Histochemical journal.* 1975;7(3):259-266. doi:10.1007/bf01003594

3. Nygaard E, Sanchez J. Intramuscular variation of fiber types in the brachial biceps and the lateral vastus muscles of elderly men: how representative is a small biopsy sample? *The Anatomical record.* 1982;203(4):451-459. doi:10.1002/ar.1092030404

4. Garrett WE, Jr., Califf JC, Bassett FH, 3rd. Histochemical correlates of hamstring injuries. *The American journal of sports medicine.* 1984;12(2):98-103. doi:10.1177/036354658401200202

5. Vikne H, Gundersen K, Liestøl K, Maelen J, Vøllestad N. Intermuscular relationship of human muscle fiber type proportions: slow leg muscles predict slow neck muscles. *Muscle & nerve.* 2012;45(4):527-535. doi:10.1002/mus.22315
